# Clinical validation of a non-heteronormative version of the Social Interaction Anxiety Scale (SIAS)

**DOI:** 10.1186/1477-7525-11-209

**Published:** 2013-12-19

**Authors:** Philip Lindner, Christopher Martell, Jan Bergström, Gerhard Andersson, Per Carlbring

**Affiliations:** 1 Department of Clinical Neuroscience, Karolinska Institutet, Karolinska, Sweden; 2 Martell Behavioral Activation Research Consulting and Department of Psychology, University of Wisconsin, Milwaukee, WI, USA; 3 Department of Psychology, Stockholm University, Stockholm, Sweden; 4 Department of Behavioural Sciences and Learning, Psychology, Linköping University, Stockholm, Sweden

**Keywords:** Heteronormativity, Social Interaction Anxiety Scale, Psychometric

## Abstract

**Introduction:**

Despite welcomed changes in societal attitudes and practices towards sexual minorities, instances of heteronormativity can still be found within healthcare and research. The Social Interaction Anxiety Scale (SIAS) is a valid and reliable self-rating scale of social anxiety, which includes one item (number 14) with an explicit heteronormative assumption about the respondent´s sexual orientation. This heteronormative phrasing may confuse, insult or alienate sexual minority respondents. A clinically validated version of the SIAS featuring a non-heteronormative phrasing of item 14 is thus needed.

**Methods:**

129 participants with diagnosed social anxiety disorder, enrolled in an Internet-based intervention trial, were randomly assigned to responding to the SIAS featuring either the original or a novel non-heteronormative phrasing of item 14, and then answered the other item version. Within-subject, correlation between item versions was calculated and the two scores were statistically compared. The two items’ correlations with the other SIAS items and other psychiatric rating scales were also statistically compared.

**Results:**

Item versions were highly correlated and scores did not differ statistically. The two items’ correlations with other measures did not differ statistically either.

**Conclusions:**

The SIAS can be revised with a non-heteronormative formulation of item 14 with psychometric equivalence on item and scale level. Implications for other psychiatric instruments with heteronormative phrasings are discussed.

## Introduction

Heteronormativity refers to practices that both originate in and reproduce presumptions and norms of there being two distinct, opposite sexes–men and women–and that these two sexes are attracted to each other (i.e. heterosexuality) without considering other sex-attraction constellations, e.g. same-sex or attraction independent of sex [1]. Despite recent changes in Western societal attitudes and practices towards non-heterosexual orientations and the individuals identified therewith (referred to as sexual minorities) [2], research suggests that sexual minorities may still experience what they consider to be inappropriate heteronormative responses from the medical-clinical community [3,4]. Heteronormative behaviours may perplex sexual minority patients resulting in communication problems, or worse, offend or embarrass sexual minority patients, alienating them from healthcare services [4-6]. Identifying heteronormative practices and replacing them with inclusive ones should therefore be made a priority for all healthcare based upon patient equality.

The increased risk of being ostracised and harassed by others has been proposed as an explanation for the finding that sexual minority individuals are at greater risk for developing psychiatric disorders [7-10]. Specifically related to their psychosocial situation, sexual minority individuals appear to show increased levels of social anxiety [6,11], i.e. a fear of negative evaluations and rejection in social situations [12]. These findings stress the importance of having screening instruments for social anxiety, validated for use with sexual minority individuals in a clinical setting.

A commonly used self-rating scale in screening for social anxiety is the Social Interaction Anxiety Scale (SIAS), developed by Mattick and Clarke [13]. The SIAS assesses fears associated with social interactions, such as sounding stupid and being ignored [13]. Item 14 of the SIAS aims to capture the anxiety associated with communicating with an attractive individual who–on the basis of sex–could be a potential partner. Unfortunately, the current phrasing of this item assumes a heterosexual orientation of the respondent: “I have difficulty talking to attractive persons of the opposite sex” [13]. For individuals who do not equate the trait of attractiveness with the opposite sex, the current phrasing creates a forced arbitrary choice of rating the difficulty of talking to either attractive people, or people of the opposite sex. This ambiguity of what is measured may lower the validity of the item and scale when used by sexual minority respondents. The current heteronormative phrasing may also insult, embarrass or demoralise sexual minority respondents, constituting an ethical problem in administering the instrument. Indirect evidence suggests that simply omitting the words “of the opposite sex” (as done in one study [11]) may yield a different interpretation of the item. While male and female ratings of male and female attractiveness tend to correlate well, heterosexual ratings of the opposite sex are more likely to include a judgment on the desirability as a potential romantic and/or sexual partner [14]. Accordingly, the original heteronormative phrasing of item 14 of the SIAS likely reflects an attempt to capture the difficulty of talking to an attractive potential partner, and not merely someone attractive. A novel, non-heteronormative phrasing of item 14 should reflect this important aspect.

Of note, none of the shortened versions of the SIAS that have been independently developed through item and factor analysis of the original instrument, have included the original item 14 [15-18]. However, there are several justifications for the continued use of the original version. The full SIAS remains popular in both clinical practice and research and using the full SIAS ensures comparability with previous findings and practices (e.g. clinical cut-offs). Further, the screening accuracies of these novel instrument versions have yet to be adequately researched, or compared. When used in research settings, were a greater span of possible results enables a greater power to detect significant differences, the original 20-item SIAS version should likewise be preferred. Hence, there are compelling reasons to continue using the full SIAS version, which requires revising the problematic 14th item with a non-heteronormative phrasing.

The purpose of this psychometric study was to develop and validate such a phrasing in a clinical sample. Our hypothesis was that our novel, non-heteronormative phrasing would yield psychometric properties similar to the original phrasing.

## Material and methods

### Participants

Participants were 129 individuals enrolled in a larger, two-phase randomised controlled trial featuring an Internet-administered attention bias modification program (ABM) and therapist-guided cognitive behavioural self-help for social anxiety disorder [19,20]. Demographic and clinical characteristics are presented in Additional file 1: Table S1 in the Supplementary material. All participants included in the intervention study fulfilled diagnostic criteria for social anxiety disorder (SAD) at initial screening, as assessed by a Structured Clinical Interview for DSM-IV axis I disorder (SCID; [21]), conducted by telephone [22]. The intervention study was approved by the Swedish Ethical Review Board (2012-132-31Ö) and pre-registered in the Clinicaltrials.gov registry (NCT01570400). See the published study protocol [19] or the primary results of the trial [20] for more details.

### Procedure

The data collection for this study was done after the two initial weeks of ABM, before the nine-week, therapist-assisted cognitive behavioural self-help program commenced. Of note, unlike in the primary analysis of the trial [20] which excluded participants who had commenced parallel treatments (n = 120), we used all available data from this measurement point (n = 129). The group assignments used in the primary analysis of the trial were not used in the present study.

After their last session of ABM, the participants were asked to once again fill out the same rating scales (including the SIAS) as was done at screening, after which the cognitive behavioural therapy would commence. Before answering the SIAS questions, participants were asked whether their birthday was on an odd or even day. This divided participants into two groups: one group that responded to the SIAS with the original item 14 (“the Original item 14 first-group”), and one group that responded to the SIAS with the novel item 14 (“the Revised item 14 first-group”). This method of randomisation was chosen because it was (a) technically feasible; (b) since it should in theory create a half-half division; and (c) because there is no reason–theoretical or empirical–to suspect that the parity of one’s birthday has any influence whatsoever on the factors relevant for this study. Having responded to the complete SIAS version to which they were randomised, all respondents were then asked the other version of item 14. Thus, all participants responded to both versions of item 14, allowing within-subject analyses. Splitting the sample into two groups was done to enable investigating effects of administration order (methods and results described in the Supplementary material). All calculations described in the main manuscript were performed on the sample undivided.

### Instruments

All measurements were made using a dedicated, secure online interface. Previous psychometric research has validated the Internet-administration of the measures used in this study [23-26] with the presentation format of one item per page [27]. All items were mandatory to ensure no missing data.

The Social Interaction Anxiety Scale (SIAS; [13]) uses 20 items and a 5-point Likert scale (scored 0–4) with verbal descriptors to assess to what degree the respondent agrees with a statement related to interaction-related characteristics of social anxiety. The SIAS has demonstrated good internal consistency, test-retest reliability, convergent validity and diagnostic accuracy [13,28,29]. The revised, non-heteronormative phrasing of SIAS item 14 was developed by authors PL and PC to capture the difficulty of talking to an attractive potential partner, without a heteronormative assumption regarding the sexual orientation of the respondent. The final wording settled upon was: “I have difficulty talking to attractive persons of the sex/sexes that I am interested in” (translation from Swedish). The phrasing allows for respondents of all sex–attraction constellations to answer the question without excluding the potential partner connotation of the original version yet remaining semantically similar to the original phrasing (“I have difficulty talking to attractive persons of the opposite sex”; see Discussion below). Comparable readability for the two item versions was assessed using the Flesch Reading Ease and Flesch-Kincaid Grade Level tests (as implemented in Microsoft Word 2010). These scores were similar: reading ease score for the revised phrasing was 47.8, compared to a score of 41.8 for the original phrasing (with higher scores indicating easier reading), while grade level score for the revised phrasing was 10.5, compared to a score of 10.1 for the original phrasing.

Other instruments administered at the same occasion provided additional measures of mental health and quality of life: the self-rated Liebowitz Social Anxiety Scale (LSAS-SR; [30,31]), the Social Phobia Scale (SPS; [13]), the self-rated Montgomery-Åsberg Depression Rating Scale (MADRS-S; [32]) and the Quality of Life Inventory [33-35]. Participants also provided free-text answers to a question on the importance of the other person’s sex on experienced social anxiety (see Additional file 1). This was done to confirm the importance of keeping the potential partner connotation in our revised phrasing of item 14.

### Calculations and analyses

Statistical analyses were performed using IBM SPSS version 22 and the R statistical environment (http://www.r-project.org), running on Windows 7. Following the recommendations outlined by Carifio and Perla [36], scale-level data was treated as interval-level data (allowing parametric tests) while individual items were not.

Psychometric equivalence of the two item versions was assessed using direct statistical comparison with the Wilcoxon Signed Ranks test and calculating the Spearman’s rank correlation between the two item versions. We also calculated the correlations between each item version and all other SIAS items to assess whether the internal consistency would differ. Both item versions were also correlated with the item-corrected total SIAS score (excluding any version of item 14 to avoid statistical dependency) and the other self-rating scales to ensure similar convergent validities. The two items’ correlation coefficients were then statistically compared using Steiger’s Z test [37], as implemented in the *cocor* R package (http://cran.r-project.org/package=cocor) [38].

Additionally, item- and scale-level data for the two groups were compared to investigate possible effects of administration order. Further details and results are described in the Supplementary material. Also included in the Supplementary material are methods and results pertaining to the analyses of free-text answers regarding the impact of sex on social anxiety. Scale-level data (total scores and internal consistencies) for the SIAS (either with the original (w/o_14_) or revised (w/r_14_) item 14 included) and the other scales are also included in the Supplementary material, both for the sample undivided and group-wise.

## Results

### Equivalence of items

The median difference in scores between the two item 14 versions was 0, with the large majority of respondents (n = 99) giving the same answer to both item versions (i.e. no difference in scores). The Wilcoxon Signed Ranks test was convincingly insignificant (*p =* 0.87). The correlation between item versions was r_s_ = 0.912. Steiger’s Z test revealed no differences in correlations to other measures between the two item 14 versions (all *p* > 0.117). See Table 1 for details.

**Table 1:** Comparison of correlation coefficients

**Measure**	**Correlation with original item 14**	**Correlation with revised item 14**	**Statistical comparison of correlation coefficients**	
			**z**	***p***
Original SIAS item 14		.912**		
Revised SIAS item 14	.912**			
Item-corrected SIAS	.497**	.479**	0.556	0.578
SIAS item 1	.424**	.394**	0.884	0.377
SIAS item 2	.487**	.454**	1.007	0.314
SIAS item 3	.236**	.241**	−0.138	0.890
SIAS item 4	.404**	.413**	−0.265	0.791
SIAS item 5	.238**	.210*	0.770	0.442
SIAS item 6	.442**	.389**	1.567	0.117
SIAS item 7	.316**	.298**	0.507	0.612
SIAS item 8	.449**	.414**	1.044	0.297
SIAS item 9	.231**	.215*	0.440	0.660
SIAS item 10	.399**	.379**	0.583	0.560
SIAS item 11	.312**	.296**	0.450	0.652
SIAS item 12	.264**	.259**	0.139	0.890
SIAS item 13	.207*	.252**	−1.239	0.215
SIAS item 15	.376**	.357**	0.548	0.584
SIAS item 16	.478**	.427**	1.540	0.124
SIAS item 17	.256**	.285**	−0.808	0.419
SIAS item 18	.127	.145	−0.486	0.627
SIAS item 19	.329**	.359**	−0.858	0.391
SIAS item 20	.302**	.296**	0.169	0.866
LSAS-SR	.446**	.426**	0.598	0.550
SPS	.302**	.252**	1.395	0.163
MADRS-S	.388**	.344**	1.270	0.204
QOLI	-.296**	-.287**	−0.252	0.801

*p < 0.05, **p < 0.01. Acronyms: SIAS, Social Interaction Anxiety Scale; LSAS-SR, Liebowitz Social Anxiety Scale self-rated; SPS, Social Phobia Scale; MADRS-S, Montgomery-Åsberg Depression Rating Scale self-rated; QOLI, Quality of Life Inventory.

### Additional analyses

The two administration-order groups did not differ in any item 14 scores, suggesting no effect of administration order. A majority of respondents (n = 84) answered “Yes” to the question “Do you find it more difficult talking to an attractive person if this person, on the basis of sex, is a potential partner?” and these respondents reported higher values on both item 14 versions. Viz., potential partner connotation appears to influence self-perceived social anxiety, at least for some. See the Additional file 1 for detailed results.

## Discussion

Heteronormativity in psychiatric and psychological practice is an under-discussed topic that is receiving increasing attention. The present study was undertaken to examine whether it was possible to revise the phrasing of item 14 of the Social Interaction Anxiety Scale (SIAS) into a non-heteronormative one that would not insult, alienate or confuse sexual minority respondents, with retained psychometric properties. Nothing in our results suggests that our novel SIAS item 14 performed differently to the original item version. We therefore conclude that the SIAS can be revised with our non-heteronormative version of item 14 without loss of psychometric compatibility with previous research and clinical practice (e.g. clinical cut-offs) derived from use of the original SIAS.

Our findings are in line with those of Weiss et al. [39] who explored four alternative rephrased versions of the same SIAS item 14. The phrasing “I have difficulty talking to a potential romantic partner” was chosen as the preferred revised version due to the near-exact equivalence of scores. Out of the three other options explored–“… an attractive person”, “… someone I could date” and “… someone I’m attracted to”–only the latter differed significantly (*p* < 0.001) in scores to the original item. Semantically, our novel phrasing, “I have difficulty talking to attractive persons of the sex/sexes that I am interested in“, can be considered to fall in-between of Weiss et al.’s preferred and discouraged alternatives, incorporating both the potential partner connotation (although implicitly) and the attractiveness connotation. Our rationale for not making the potential partner connotation explicit in our phrasing was that including the word “partner” could be interpreted to equal a committed relationship, in contrast to e.g. a causal sexual relationship. Such a possible interpretation was not supported by the original phrasing and could therefore be a potential confounder in some cultural contexts. Hence, our version could be considered semantically more similar to the original version of SIAS item 14 than Weiss et al.’s. Whether this fine difference would yield any empirical difference in scores in any cultural context remains unexplored. Aside from validating a novel phrasing of item 14, the present study also extends previous research in several important aspects. First, the present study was performed on a clinical sample: considering that the SIAS is used in clinical contexts–as an outcome measure in clinical trials ( e.g. in [19]) and for screening purposes–a psychometric evaluation featuring a clinical sample was necessary before any revised edition was to be recommended for use in such populations. The present study also featured a more diverse set of other measures of well-being to ensure similar convergent validities of item versions. The rationale behind the rephrasing was also tested, albeit relying only on self-reported experience. Importantly, this study provides some initial cross-cultural validity to the rephrasing of heteronormative items in social anxiety self-rating scales.

Unfortunately, the SIAS is far from the only psychiatric instrument that features heteronormative language. In Sweden, the gold standard in assessing obsessive-compulsive disorder (OCD), the Yale-Brown Obsessive Compulsive Scale (Y-BOCS) [40], gave rise to a debate on heteronormativity and possible discrimination of sexual minorities in psychiatry [41]. The Y-BOCS features an item that screens for sexual obsessions with “content [that] involves homosexuality”, the administration of which led to accusations of heteronormativity and pathologising homosexuality. Aside from the self-report measures of SAD noted by Weiss et al. [39], the widely used Symptom Checklist 90 [42] includes an item (number 21) similar in phrasing to SIAS item 14. Although the findings of the current study may be difficult to translate to the context of other psychiatric conditions (e.g. OCD), they do suggest that psychiatric rating scales can be revised with non-heteronormative formulations that do not confuse, insult or alienate sexual minority respondents.

This study has some limitations that should be recognised. First, the study design did not allow for investigation of test-retest reliability. While acknowledging that single-item analyses are particularly vulnerable to low test-retest reliability, other aspects of psychometric integrity in our study (e.g. internal consistencies, see Additional file 1: Table S2) were in line with previous studies that additionally showed sound test-retest reliability. Hence, we see no reason to suspect that the test-retest reliability should be lower in the current study. Second, the sample size of 129 participants should be considered only moderate, although in line with previously published psychometric evaluations by our research group (e.g. [24]). Future studies with larger and more diverse samples would enable examining differential item functioning in different groups. In this study, participants were administered all rating scales via the Internet. However, since previous research has shown that the psychometric properties of the Internet-administered SIAS are similar to the pen-and-paper version [24], the results of this study should be equally valid for paper-administered versions of the SIAS. Finally, we cannot exclude the possibility of an impact of the cultural context in which this study was performed. While our non-heteronormative formulation of item 14 does not make any explicit reference to any sexual orientation, the phrasing does invite further interpretation and hence, cultural factors may be invoked. This study was conducted in Sweden, a country that relative to many other countries enjoys increasing acceptance and recognition of sexual minority individuals. Therefore the present study would benefit from replication in other languages and cultures.

## Conclusion

This study concludes that the Social Interaction Anxiety Scale (SIAS) can be revised with the non-heteronormative formulation of item 14 herein described without affecting the psychometric properties. This non-heteronormative revision of SIAS is recommended for use in both clinical and research settings to avoid potentially alienating, confusing or insulting sexual minority respondents.

## Abbreviations

ABM: Attention bias modification training; LSAS-SR: Liebowitz social anxiety scale self-rated; MADRS-S: Montgomery-Åsberg depression rating scale self-rated; SIAS: Social interaction anxiety scale; SPS: Social phobia scale; QOLI: Quality of life inventory.

## Competing interests

The authors declare that they have no competing interests.

## Authors’ contributions

Authors PL and PC designed the study. PL carried out analyses and drafted the manuscript. JB carried out additional analyses. CM, JB, GA and PC made significant contributions to the study rationale and interpretation of findings. All authors participated in the review and revision of the manuscript and have approved the final manuscript to be published.

## Supplementary Material

Additional file 1: Table S1 Demographics and clinical characteristics of the sample and between-group statistical comparisons.**Table S2.** Internal consistencies (Cronbach’s alphas) of all scales. **Table S3.** Matrix of correlation coefficients for each group seperately. **Figure S1.** Relative distributions of responses to the revised and original SIAS item 14 between those who answered “Yes” and “No” to the question “Do you find it more difficult talking to an attractive person if this person, on the basis of sex, is a potential partner?” Click here for file
