# Supplementary material for: Clinical validation of a non-heteronormative version of the Social Interaction Anxiety Scale (SIAS)
Source: Health Qual Life Outcomes. 2013 Dec 19;11:209. doi: 10.1186/1477-7525-11-209 (PMC3878259; doi:10.1186/1477-7525-11-209)
Supplement: Additional file 1: Table S1 — Demographics and clinical characteristics of the sample and between-group statistical comparisons.Table S2. Internal consistencies (Cronbach’s alphas) of all scales. Table S3. Matrix of correlation coefficients for each group seperately. Figure S1. Relative distributions of responses to the revised and original SIAS item 14 between those who answered “Yes” and “No” to the question “Do you find it more difficult talking to an attractive person if this person, on the basis of sex, is a potential partner?” [file 1477-7525-11-209-S1.pdf]

Supplementary material to: Clinical validation of a non-heteronormative version of the Social Interaction Anxiety Scale (SIAS)

Philip Lindner[1*]	philip.lindner@ki.se
Christopher Martell[2]	christophermartellphd@gmail.com
Jan Bergström[3]	jan.bergstrom@psychology.su.se
Gerhard Andersson[1,4]	gerhard.andersson@liu.se
Per Carlbring[3]	per@carlbring.se

[1] Dep. of Clinical Neuroscience, Karolinska Institutet, Sweden
[2] Martell Behavioral Activation Research Consulting and Dep. of Psychology, University of Wisconsin, Milwaukee, WI, USA
[3] Dep. of Psychology, Stockholm University, Sweden
[4] Dep. of Behavioural Sciences and Learning, Psychology, Linköping University, Sweden

[*] Corresponding author.

Psychiatry building R5:00
Karolinska University Hospital Solna
17176, Stockholm, Sweden

Supplementary methods

Participants

Demographics and clinical characteristics of the sample are presented in Table S1.

Additional instruments

Liebowitz Social Anxiety Scale self-rated (LSAS-SR)

The self-rated Liebowitz Social Anxiety Scale (LSAS-SR; [1, 2]) measures both fear in, and avoidance of 24 social situations (totalling 48 items) using a 4-point scale (scored 0-3) with verbal descriptors. Originally developed as clinician-administered instrument, the self-rated version has since become popular and exhibits similar psychometric properties to the original [3]. A strong correlation (r = 0.85) between the two administration formats has also been reported [3]. The LSAS-SR has demonstrated good internal consistency, test-retest reliability, convergent and discriminative validity and sensitivity to change [1, 3]. Consistent with previous clinical and psychometric research by our research group [4, 5], we report here a single LSAS-SR score and not the fear and avoidance scale scores separately.

Social Phobia Scale (SPS)

Like its companion instrument the SIAS, the Social Phobia Scale (SPS; [6]) measures social anxiety using 20 items and a 5-point Likert scale (scored 0-4) with verbal descriptors. The SPS assesses fears of being scrutinised by others during everyday activities such as using public transportation, and has demonstrated good internal consistency, test-retest reliability, convergent validity and diagnostic accuracy [6, 7]. Factor analysis has provided psychometric support that the SPS and SIAS measure similar yet distinct constructs of social anxiety [8, 9].

Montgomery-Åsberg Depression Rating Scale self-rated (MADRS-S)

The self-rated Montgomery-Åsberg Depression Rating Scale (MADRS-S; [10]) was used to measure symptoms of depression, known to commonly co-occur with SAD. The MADRS-S consists of 9 items corresponding to depressive symptoms, rated on item-unique 7-point scales, all with 4 verbal descriptors and 3 non-defined steps in-between. Good internal consistency, test-retest reliability and correlation with the clinician-administered version has been demonstrated [10, 11].

Quality of Life Inventory (QOLI)

The self-rated Quality of Life Inventory [12–14] assesses quality of life across 16 domains, including work, health, recreation and other important aspects of life not captured by pathology-oriented rating scales, making it a meaningful complement when assessing clinical severity. Unique for the QOLI is that the respondent not only rates satisfaction (score ±1-3) in each area, but also weighs each area by multiplying the satisfaction rating with the perceived importance (scored 0-2) of this area for overall quality of life (equalling 16 × 2 = 32 items). An average quality of life score is calculated by averaging all weighted, non-zeroed ratings, according to standard scoring procedure.

The impact of sex on social anxiety

After completing all self-rating scales, participants also provided a free-text answer to the question: "Do you find it more difficult talking to an attractive person if this person, on the basis of sex, is a potential partner?" (translation from Swedish). Of note, what is measured here is self-reported, self-perceived effect of sex on social interaction anxiety.

Additional calculations and analyses

Effects of administration order

To test whether there was any effect of administration order, we compared item 14 scores and discrepancies for the two groups (“Original item 14 first-group” and the “Revised item 14 first-group”). To ensure that the two groups were similar in all other relevant aspects, we also statistically compared demographics and scale total scores. Group-wise internal consistencies and correlation coefficients were qualitatively compared.

Potential partner connotation

Free-text answers to the question “Do you find it more difficult to talking to an attractive person if this person, on the basis of sex, is a potential partner?” were reviewed and rated categorically (Yes/No) by author PL. Author JB reviewed and rated the same answers independently and inter-rater reliability was calculated with Cohen’s kappa. Analyses of the effects of sex on social anxiety were performed on the sample undivided.

Supplementary results

Sample characteristics and effects of administration order

Demographic and clinical characteristics are presented in Table S1 below. Internal consistencies and correlation coefficients were overall very similar across groups and are displayed in Table S2 and S3 (respectively) below. The two groups did not differ in scores on either the original or revised item 14 (Mann-Whitney U tests $p > 0.159$) or in distribution of discrepancies between item 14 version scores ($\chi^2 = 3.890$, $p = 0.411$). F and χ^2 tests revealed no significant between-group differences in other clinical or demographic variables (see Table S1). However, for reasons unknown, the sex distribution did show a near-significant difference. To ensure that any between-group difference in SIAS item 14 scores was not due

to the skewed sex distribution, we ran a Mann-Whitney *U* test on the sample undivided with sex as the independent variable and the original and revised item 14 scores as the dependent variables. No significant ($p > 0.69$) between-sex differences were found, indicating that the near-significant between-group difference in sex distribution should not impact between-group comparisons of item 14 scores.

Potential partner connotation

The ratio of deemed Yes/No answers to question “Do you find it more difficult talking to an attractive person if this person, on the basis of sex, is a potential partner?” was found to be 70.6/29.4% (n = 84/35). The answers of ten individuals were inconclusive, contradictory or uninformative and could hence no be classified. Inter-rater reliability (Cohen’s kappa) was calculated to 0.783 ($p < 0.001$). Additional analyses were conducted to test whether the answer (Yes/No) to this question had any impact on other metrics. *F* tests revealed no significant differences between the Yes and No groups on the LSAS-SR, SPS, MADRS-S, QOLI or two SIAS versions (either w/o_{14} or w/r_{14}). However, Mann-Whitney *U* tests revealed significant ($p < 0.001$) between-group (Yes/No) differences in responses to both the revised and original versions of item 14. In both cases: the Yes group > No group. See Figure S1 for distribution of responses between the Yes and No groups on both item versions. In essence, those who answered Yes to the question “Do you find it more difficult talking to an attractive person if this person, on the basis of sex, is a potential partner?”, reported higher values on both item 14 versions, but on a scale level, there was no difference. Using the inter-rater assessment of Yes/No answers as the grouping variable did not alter any finding across any of the *p*-levels.

References

1. Baker SL, Heinrichs N, Kim H-J, Hofmann SG: **The liebowitz social anxiety scale as a self-report instrument: a preliminary psychometric analysis.** *Behav. Res. Ther.* 2002, **40**:701–15.

2. Liebowitz MR: **Social phobia.** *Mod. Probl. Pharmacopsychiatry* 1987, **22**:141–173.

3. Fresco DM, Coles ME, Heimberg RG, Liebowitz MR, Hami S, Stein MB, Goetz D: **The Liebowitz Social Anxiety Scale: a comparison of the psychometric properties of self-report and clinician-administered formats.** *Psychol. Med.* 2001, **31**:1025–35.

4. Hedman E, Ljótsson B, Rück C, Furmark T, Carlbring P, Lindefors N, Andersson G: **Internet administration of self-report measures commonly used in research on social anxiety disorder: A psychometric evaluation**. *Comput. Human Behav.* 2010, **26**:736–740.

5. Boettcher J, Hasselrot J, Sund E, Andersson G, Carlbring P: **Combining Attention Training with Internet-Based Cognitive-Behavioural Self-Help for Social Anxiety: A Randomised Controlled Trial.** *Cogn. Behav. Ther.* 2013:37–41.

6. Mattick RP, Clarke JC: **Development and validation of measures of social phobia scrutiny fear and social interaction anxiety.** *Behav. Res. Ther.* 1998, **36**:455–70.

7. Peters L: **Discriminant validity of the Social Phobia and Anxiety Inventory (SPAI), the Social Phobia Scale (SPS) and the Social Interaction Anxiety Scale (SIAS)**. *Behav. Res. Ther.* 2000, **38**:943–950.

8. Heidenreich T, Schermelleh-Engel K, Schramm E, Hofmann SG, Stangier U: **The factor structure of the Social Interaction Anxiety Scale and the Social Phobia Scale.** *J. Anxiety Disord.* 2011, **25**:579–83.

9. Carleton RN, Collimore KC, Asmundson GJG, McCabe RE, Rowa K, Antony MM: **Refining and validating the Social Interaction Anxiety Scale and the Social Phobia Scale.** *Depress. Anxiety* 2009, **26**:E71–81.

10. Svanborg P, Åsberg M: **A new self-rating scale for depression and anxiety states based on the Comprehensive Psychopathological Rating Scale**. *Acta Psychiatr. Scand.* 1994, **89**:21–28.

11. Bondolfi G, Jermann F, Rouget BW, Gex-Fabry M, McQuillan A, Dupont-Willemin A, Aubry J-M, Nguyen C: **Self- and clinician-rated Montgomery-Asberg Depression Rating Scale: evaluation in clinical practice.** *J. Affect. Disord.* 2010, **121**:268–72.

12. Frisch MB, Cornell J, Villanueva M, Retzlaff PJ: **Clinical validation of the Quality of Life Inventory. A measure of life satisfaction for use in treatment planning and outcome assessment.** *Psychol. Assess.* 1992, **4**:92–101.

13. Paunović N, Öst L-G: **Clinical Validation of the Swedish Version of the Quality of Life Inventory in Crime Victims with Posttraumatic Stress Disorder and a Nonclinical Sample**. *J. Psychopathol. Behav. Assess.* 2004, **26**:15–21.

14. Frisch MB, Clark MP, Rouse S V, Rudd MD, Paweleck JK, Greenstone A, Kopplin D a: **Predictive and treatment validity of life satisfaction and the quality of life inventory.** *Assessment* 2005, **12**:66–78.

Table S1.
Demographics, clinical characteristics and between-group differences.

Variable	Sample undivided (n=129)	Original item 14 first-group (n=55)	Revised item 14 first-group (n=74)	Between-group statistics
**Mean age** (SD)	33.8 (10.3)	34.4 (10.2)	33.3 (10.4)	*F* = 0.400 (*p* = 0.53)
**Sex** (m/f)	45/84	14/41	31/43	χ^2 = 3.753 (*p* = 0.063)
**Marital status**				χ^2 = 2.041 (*p* = 0.927)
Married W/C:	34	14	20	
Married N/C:	30	14	16	
LAT W/C:	3	1	2	
LAT N/C:	7	4	3	
Single W/C:	11	4	7	
Single N/C:	41	16	25	
Other:	3	2	1	
**Education**				χ^2 = 4.270 (*p* = 0.537)
Primary UF:	1	0	1	
Primary:	3	1	2	
Secondary:	19	8	11	
PS:	13	3	10	
Current university:	35	14	21	
Finished university:	58	29	29	
**Prior psychological treatment**				χ^2 = 3.109 (*p* = 0.212)
No:	63	28	35	
Yes, prior:	64	25	39	
Yes, current:	2	2	0	
**Prior pharmacological treatment**				χ^2 = 1.166 (*p* = 0.586)
No:	75	29	46	
Yes, prior:	37	18	19	
Yes, current:	17	8	9	
**Currently on sick-leave** (yes/no)	4/125	2/53	2/72	χ^2 = 0.092 (*p* = 1.0)
**SIAS w/o$_{14}$** (SD)	48.40 (15.63)	50.15 (15.88)	47.09 (15.42)	*F* = 1.204 (*p* = 0.275)
**SIAS w/r$_{14}$** (SD)	48.39 (15.59)	50.09 (15.86)	47.12 (15.37)	*F* = 1.146 (*p* = 0.286)

**Item-corrected SIAS** (SD)	46.02 (14.89)	47.56 (15.25)	44.86 (14.61)	*F* = 1.037 (*p* = 0.310)
**SPS** (SD)	35.39 (14.08)	35.69 (15.21)	35.16 (13.28)	*F* = 0.044 (*p* = 0.834)
**LSAS-SR** (SD)	67.19 (22.57)	69.53 (24.69)	65.45 (20.85)	*F* = 1.032 (*p* = 0.312)
**MADRS-S** (SD)	12.99 (7.35)	13.31 (8.63)	12.76 (6.29)	*F* = 0.177 (*p* = 0.675)
**QOLI** (SD)	0.99 (1.70)	1.11 (1.76)	0.91 (1.67)	*F* = 0.449 (*p* = 0.504)

Additional acronyms used: LAT, living apart together; W/C, with children; N/C, no children; SD, standard deviation; UF, unfinished; PS, professional school.

Table S2.
Internal consistencies (Cronbach’s alphas).

Measure	Sample undivided (n=129)	The Original item 14 first-group (n=55)	The Revised item 14 first-group (n=74)
SIAS w/o_{14}	0.924	0.933	0.917
SIAS w/r_{14}	0.924	0.933	0.918
Item-corrected SIAS	0.923	0.933	0.914
SPS	0.894	0.910	0.879
LSAS-SR	0.949	0.958	0.941
MADRS-S	0.843	0.886	0.787
QOLI	0.826	0.826	0.826

Table S3.

Matrix of correlation coefficients. Upper panel show the Original item 14 first-group, lower panel shows the Revised item 14 first-group.

	SIAS14org	SIAS14rev	Item-corrected SIAS	SIAS w/o14	SIAS w/r14	LSAS-SR	SPS	MADRS-S	QOLI
SIAS14org		.902**	.462**		.525**	.433**	.340*	.483**	-.328*
SIAS14rev	.925**		.451**	.510**		.444**	.277*	.435**	-.285*
Item-corrected SIAS	.544**	.534**				.783**	.649**	.634**	-.456**
SIAS w/o14		.605**				.789**	.651**	.647**	-.464**
SIAS w/r14	.611**					.792**	.647**	.644**	-.459**
LSAS-SR	.445**	.405**	.758**	.759**	.756**		.756**	.714**	-.557**
SPS	.312**	.277*	.648**	.642**	.640**	.731**		.643**	-.404**
MADRS-S	.299**	.252*	.485**	.489**	.485**	.432**	.433**		-.707**
QOLI	-.297*	-.310**	-.310**	-.320**	-.320**	-.175	-.183	-.462**	

* $p < 0.05$, ** $p < 0.01$. Statistically dependent correlations are blanked.

Figure S1.

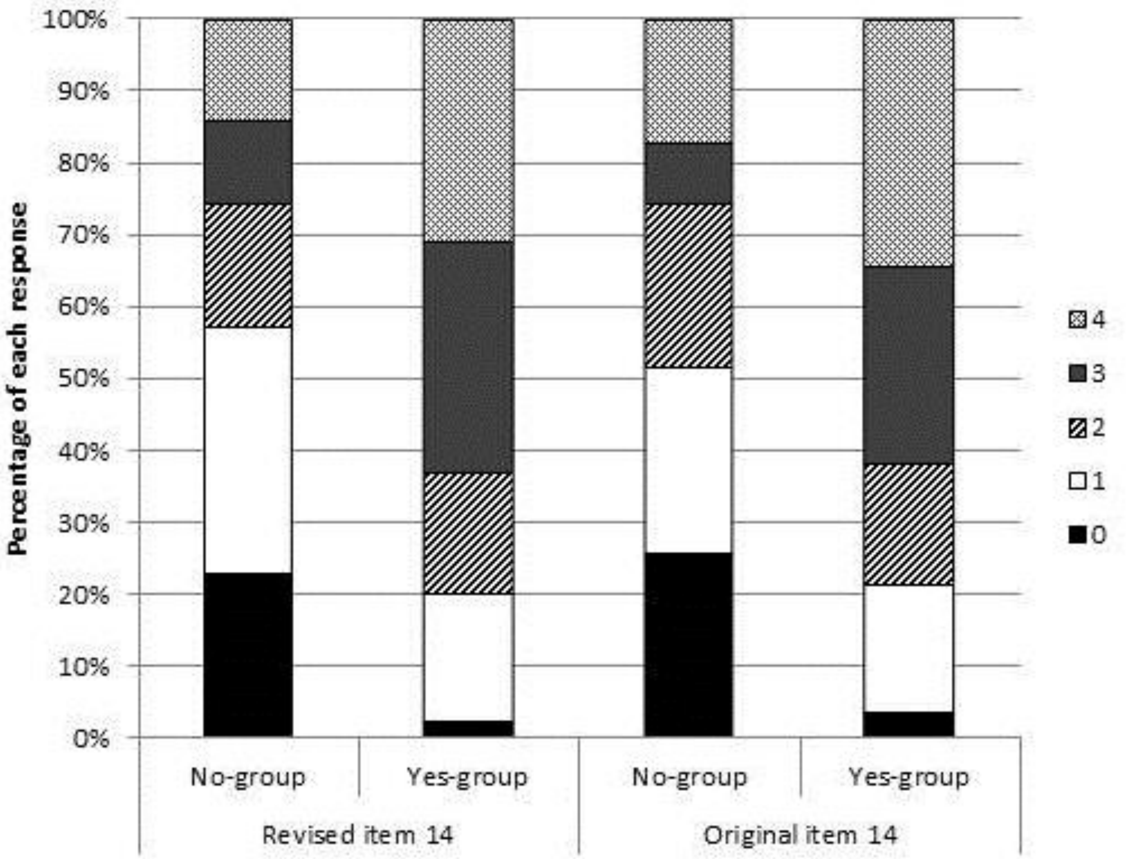

Relative distributions of responses to the revised and original SIAS item 14 between those who answered “Yes” and “No” to the question “Do you find it more difficult talking to an attractive person if this person, on the basis of sex, is a potential partner?”
